# Molecular Evidence of Novel Spotted Fever Group *Rickettsia* Species in *Amblyomma albolimbatum* Ticks from the Shingleback Skink (*Tiliqua rugosa*) in Southern Western Australia

**DOI:** 10.3390/pathogens10010035

**Published:** 2021-01-05

**Authors:** Mythili Tadepalli, Gemma Vincent, Sze Fui Hii, Simon Watharow, Stephen Graves, John Stenos

**Affiliations:** 1 Australian Rickettsial Reference Laboratory, University Hospital Geelong, Geelong 3220, Australia; mythili.tadepalli@barwonhealth.org.au (M.T.); GEMMA.VINCENT@barwonhealth.org.au (G.V.); sze.hii@unimelb.edu.au (S.F.H.); graves.rickettsia@gmail.com (S.G.); 2 Reptile Victoria Inc., Melbourne 3035, Australia; smwatharow@gmail.com; 3 Department of Microbiology and Infectious Diseases, Nepean Hospital, NSW Health Pathology, Penrith 2747, Australia

**Keywords:** *Rickettsia*, infectious diseases, reptile, molecular epidemiology

## Abstract

Tick-borne infectious diseases caused by obligate intracellular bacteria of the genus *Rickettsia* are a growing global problem to human and animal health. Surveillance of these pathogens at the wildlife interface is critical to informing public health strategies to limit their impact. In Australia, reptile-associated ticks such as *Bothriocroton hydrosauri* are the reservoirs for *Rickettsia honei*, the causative agent of Flinders Island spotted fever. In an effort to gain further insight into the potential for reptile-associated ticks to act as reservoirs for rickettsial infection, *Rickettsia*-specific PCR screening was performed on 64 *Ambylomma albolimbatum* ticks taken from shingleback skinks (*Tiliqua rugosa*) located in southern Western Australia. PCR screening revealed 92% positivity for rickettsial DNA. PCR amplification and sequencing of phylogenetically informative rickettsial genes (*ompA, ompB, gltA, sca4,* and *17kda*) suggested that the single rickettsial genotype detected represented a novel rickettsial species, genetically distinct from but closely related to *Rickettsia gravesii* and within the rickettsia spotted fever group (SFG). On the basis of this study and previous investigations, it would appear that *Rickettsia* spp. are endemic to reptile-associated tick species in Australia, with geographically distinct populations of the same tick species harboring genetically distinct SFG *Rickettsia* species. Further molecular epidemiology studies are required to understand the relationship between these diverse *Rickettsiae* and their tick hosts and the risk that they may pose to human and animal health.

## 1. Introduction

Ticks are important vectors of infectious disease in humans and animals [1]. Ticks preceded mosquitos as being the first arthropods ever associated with the transmission of infection to a healthy vertebrate animal (Texas cattle fever), as determined by Theobald Smith and colleagues in 1889. Of the most important groups of pathogens transmitted by ticks, the obligate intracellular bacteria of the order Rickettsiales are paramount. Knowledge of this group of bacteria has been transformed recently with the advent of molecular techniques, leading to the description of a growing diversity of emerging tick-borne rickettsioses [2]. 

Australia has a rich diversity of hard and soft tick species, with at least 70 commonly found across the continent [3]. Several of these species have been introduced, although the majority are unique to Australia [3]. It is perhaps not surprising that these ticks are reservoirs for a variety of novel *Rickettsia* species not described elsewhere, including rickettsiae recognized to cause infectious diseases in humans following a tick bite [4]. 

Five rickettsial species/subspecies have been described as human pathogens in Australia. The first three are tick-transmitted, while the remainder are flea-transmitted. These rickettsiae include (i) *Rickettsia australis*, the causative agent of Queensland tick typhus and the first rickettsiae isolated from febrile patients in Australia [5]; (ii) *R. honei,* the etiological agent of Flinders Island spotted fever (FISF) [6], a disease localized mainly to the southern regions of Australia [7]; (iii) a subspecies of the latter species, *R. honei* subsp. *marmionii*, causing Australian spotted fever in the north and east of Australia [8]; (iv) *R. typhi*, located Australia wide in rodent fleas [9]; and (v) *Rickettsia felis*, probably Australia wide and endemic in cat fleas [10]. Two other Australian rickettsial species have been suspected of causing human infection based on their detection in ticks known to bite humans (e.g., *Candidatus Rickettsia tasmanensis* [11] and *R. gravesii* [12]).

All the Australian rickettsial species, with the exception of *Rickettsia typhi* and *Orientia* spp., belong to a broader subgroup known as the spotted fever group (SFG), with over 30 *Rickettsia* member species spread across every continent. This group cannot usually be serologically distinguished from the typhus group (TG) species in the genus *Rickettsia* [13]. They all cause an acute infection typically featuring fever, headache, and a maculopapular rash [14]. Human infection is accidental except human louse-borne epidemic typhus caused by *Rickettsia prowazekii*, which is not endemic in Australia. Various wildlife species are considered competent hosts, and in Australia, these are suspected to include native fauna such as marsupials [11,15] and reptiles [16,17]. Indeed, surveillance studies have previously revealed that reptile-associated ticks (*Bothriocroton hydrosauri)* can harbor the etiological agents of FISF, *R. honei* [16]. Similarly, PCR screening of the same ticks on the Australian mainland (South Australia) found a very high (100%) rickettsial prevalence [18]. Molecular analyses, albeit limited to a single gene, suggested that the detected rickettsiae were not *R. honei* and instead were closely related to unclassified rickettsiae previously detected in Australian *Amblyomma fimbriatum* ticks from the Northern Territory, Australia [17]. 

In the current study, we have conducted expanded molecular screening for rickettsiae found in reptile-associated ticks from the southern part of Western Australia. Rickettsial species detected were subsequently classified using a molecular typing scheme previously described for rickettsial identification and classification [19]. 

## 2. Results

Ticks (*n* = 192) were sampled from 26 heavily infested shingleback skinks *(Tiliqua rugosa)* from several locations in the southern part of Western Australia during the spring of 2016. Morphological identification revealed that all ticks were *A. albolimbatum*. Tick numbers ranged from 1–19 ticks per animal.

Of the 64 *A. albolimbatum* ticks screened by real-time PCR (qPCR), 59 (92.2%) were positive for rickettsial DNA (Table 1). Amongst the different life cycle stages screened, all adult female ticks (21/21) and nymphs (4/4) as well as 34/39 (87.2%) adult male ticks returned positive results. 

**Figure 1:**
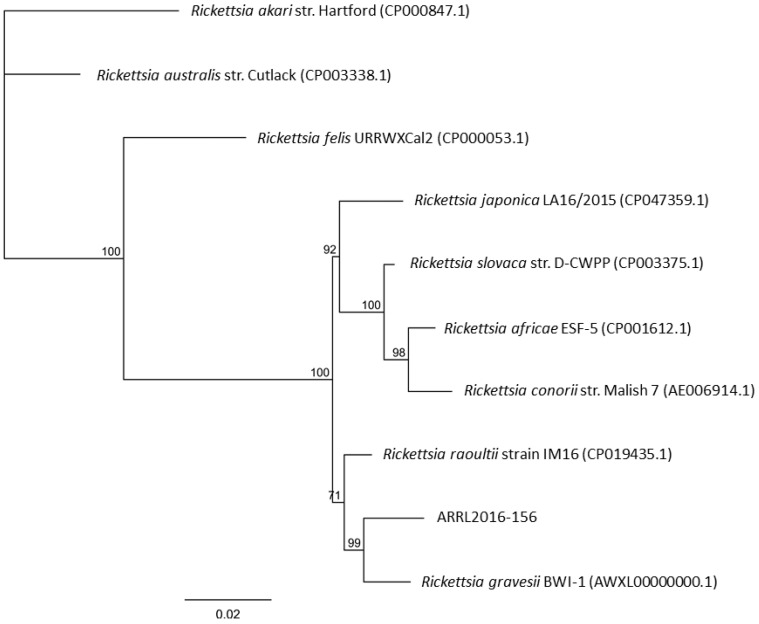
*Rickettsia* phylogenetic tree. The phylogenetic relationship between the newly detected *Rickettsia* spp. (ARRL2016-156) to other closely related rickettsiae was determined by comparing concatenated gene sequences (*gltA*, *17kda*, *ompB*, and *sca4*). Branch lengths indicate the number of substitutions per site. The percentage of trees in which the associated taxa clustered together is shown next to the branches after 100 bootstrap replications.

To molecularly identify the *Rickettsia* spp. present, additional conventional PCRs targeting the *gltA, ompB, 17kda, sca4,* and *ompA* genes were performed on samples from three ticks: ARRL2016-149, ARRL2016-156, and ARRL2016-159. Sequence analysis of the amplified PCR products from each tick sample revealed they were identical. BLAST analysis of each partial gene sequence revealed closest similarity to *Rickettsia raoultii* (*gltA*: 100%, Genbank Accession No. MN388798.1; *17kda*: 100%, MH932036.1) and *R. gravesii* (*ompB*: 97.0%, DQ269438.1; *sca4*: 97.8%, DQ269439.1). To resolve the identity of the strain detected in the study, phylogenetic trees were constructed using the concatenated gene sequences (*gltA, ompB, 17kda,* and *sca4*) of ARRL2016-156 against other species in the genus *Rickettsia* (Figure 1). This analysis revealed that the rickettsiae detected in this study belong to the *Rickettsia* SFG subgroup, clustering in a distinct subclade *R. gravesii*.

**Figure 2:**
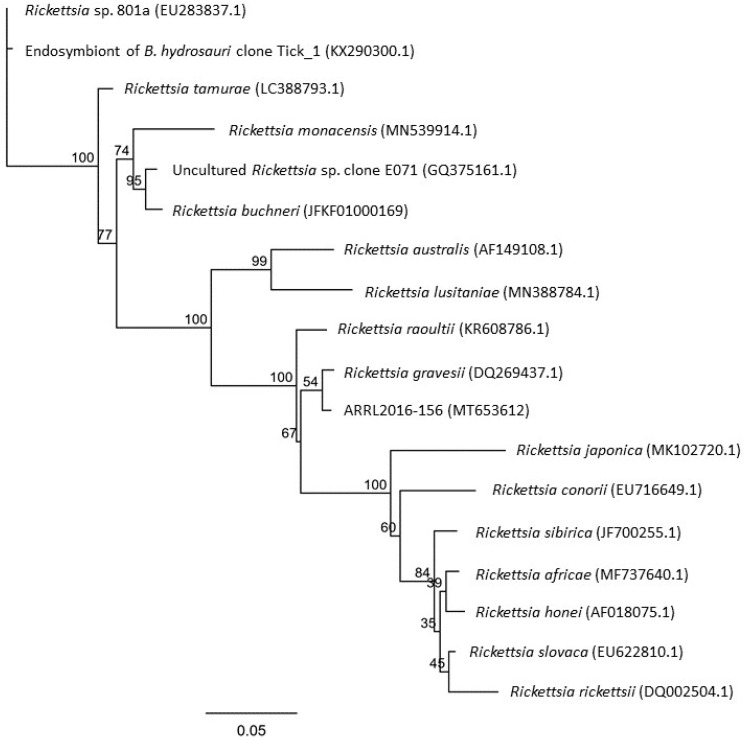
*Rickettsia ompA* gene sequence phylogenetic tree. The phylogenetic relationship between the newly detected *Rickettsia* spp. (ARRL2016-156) to other closely related rickettsiae was determined by comparing partial *ompA* gene sequences. Branch lengths indicate the number of substitutions per site. The percentage of trees in which the associated taxa clustered together is shown next to the branches after 100 bootstrap replications.

To gain further insight into the phylogenetic position of this newly detected *Rickettsia* to other species in the *Rickettsia* SFG, partial gene sequences were amplified from the *Rickettsia ompA* gene found in *Rickettsia* SFG members. PCR amplification was successful only for two tick samples (ARRL2016-156 and ARRL2016-159) with the resulting analysis confirming *ompA* sequences were identical and most closely related to sequences for *R. gravesii* (DQ269437; 98.84%). Phylogenetic analysis of the ARRL2016-156 *ompA* sequence against other *Rickettsia* SFG subgroup members confirmed that the newly detected *Rickettsiae* detected in this study clustered most closely with *R. gravesii* (Figure 2) and were genetically distinct from *ompA* sequences previously amplified from the tick *B. hydrosauri*.

## 3. Discussion

A “One Health” approach that prioritizes surveillance of ticks and wildlife, the most common reservoirs of rickettsial pathogens, is considered key in attempts to respond to the growing global burden of tick-borne diseases [20]. Molecular techniques, including PCR-based strategies [2] as well as next-generation sequencing [21], are critical for the surveillance of intracellular pathogens such as rickettsiae that cannot yet be cultured axenically. 

In the current study, molecular screening revealed a 92% PCR positivity for rickettsial DNA in *A. albolimbatum* ticks collected from the shingleback skink (*T. rugosa)* in the southern part of Western Australia. All developmental stages of *A. albolimbatum* were found to be positive. These high PCR positive results (almost 100%) were surprisingly similar to the results described in a previous study by Whiley et al. [18] investigating the positivity of rickettsiae in *B. hydrosauri* ticks removed from the same reptilian host in South Australia. Animals sampled in the latter study were located approximately 2000 km east of those sampled in this current investigation. This high rate of rickettsial positivity from Australian reptiles contrasts with recent PCR-based screening studies for the presence in ticks removed from marsupials, domesticated animals, and humans, which revealed a prevalence of only 6–15% [22,23,24]. The high incidence reported in reptile ticks may suggest that these rickettsiae exist in in an endosymbiotic relationship with their reptile tick host [25,26]. In contrast, the rickettsiae identified in mammalian tick species [22,23,24] may be simply commensals. Further work, including sampling of ticks in the field prior to feeding, is required to investigate the true prevalence and relationship of these novel rickettsiae to their tick host.

In terms of the potential impact on human and animal health, concern has existed for the presence of *Rickettsia* spp. in reptile ticks from Australia ever since *R. honei*, the causative agent of FISF, was identified in the reptile tick *B. hydrosauri* [16], indicating that tick bites caused by the latter species could transmit and cause disease in humans. The risk to human health is further emphasized by the molecular evidence suggesting that the rickettsiae detected in this study belong to the rickettsial SFG. The latter group contains a range of rickettsiae with proven pathogenic potential in humans, including *R. raoultii*, to which it is closely related. While no other human pathogens have yet been identified in ticks from reptiles, and we lack any evidence on the pathogenic potential of the rickettsia identified in this study, the high prevalence of rickettsial species in Australian reptile ticks should be noted. While we have no evidence of the pathogenic potential of the novel tick rickettsiae, efforts to raise public awareness and educating people working or engaging in recreational activities in reptile habitats on the risk of tick-borne diseases may be appropriate. 

The results of this study suggest that the molecular epidemiology of rickettsial infections in Australian reptile-associated ticks is complex, with reptilian ticks such as *B. hydrosauri* the likely reservoirs for at least three genetically distinct rickettsial species in the SFG, including (a) *R. honei* found in *B. hydrosauri* ticks, (b) the rickettsial species detected in this current study from *A. albolimbatum* ticks, and (c) rickettsial (presumed) endosymbionts found in *B. hydrosauri* removed from the same reptile (*T. rugosa*) found in another study [18]. Evidence for the latter is limited to amplification and analysis of partial *Rickettsia ompA* sequences; however, the phylogenetic analysis between these sequences and those amplified in this study (Figure 2) clearly show that they belong to different subclades within the *Rickettsia* SFG. While the phylogenetic trees reveal close clustering to *R. gravesii* [12], the new *Rickettsia* SFG discovered in this Australian tick showed sufficient nucleotide dissimilarity within the *ompA* (1.2%) and *ompB* (3.0%) sequences compared to *R. gravesii* to be considered different. Based on criteria previously proposed by Fournier et al. [19], it is possible that the *Rickettsia* spp. detected in this current study may represent a new species within the genus *Rickettsia.* The *A. albolimbatum* ticks analyzed in this study came from geographically distinct *T. rugosa* populations in the southern part of Western Australia, suggesting that the newly identified rickettsial agent may be widely distributed throughout this region. Further molecular typing, including comparative genomic analysis, and isolation is required for formal classification based on whole-genome sequencing. 

An obvious limitation of the current investigation is that molecular typing of only three ticks was performed. With appropriate resourcing, it would be possible to conduct expanded molecular epidemiological investigation into the diversity, including vertebrate and invertebrate hosts and their ranges, so as to improve understanding of pathogen transmission, host preference, and other factors that influence the presence and geographic distribution of these obligate intracellular bacteria. 

## 4. Materials and Methods 

### 4.1. Tick Collection and Identification

**Figure 3:**
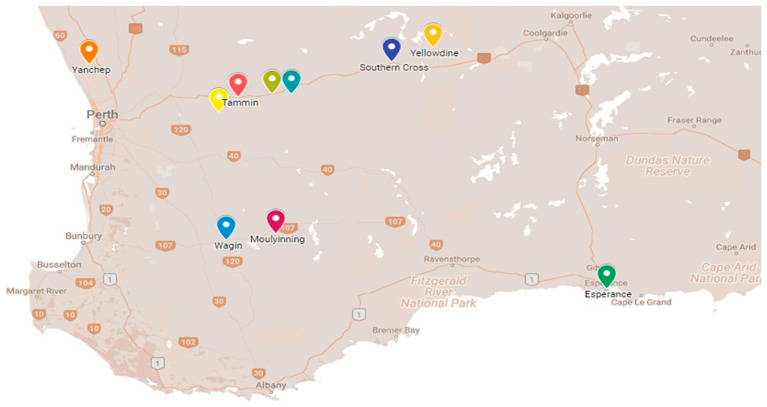
Map of southwest Australia with shingleback skink (*Tiliqua rugosa*) sampling sites indicated by colored markers. Map prepared with Google MyMaps (www.google.com/mymaps).

Ticks (*n* = 192) screened in this study were collected from shingleback skinks (*Tiliqua rugosa*) captured in October 2016 from various locations extending up to approximately 700 km west and south from Perth in the southwest corner of the Australian continent (Figure 3). Ticks were sent to the Australian Rickettsial Reference Laboratory (ARRL) for further examination. Sampled ticks were then morphologically identified according to previously published tick identification keys [27,28]. 

### 4.2. DNA Extraction and Rickettsia-Specific qPCR Detection

After identification, 64 ticks from this larger collection were individually cleaned with phosphate-buffered saline (PBS), dissected, and homogenized using a micropestle in 300 µL of PBS. DNA was then extracted from 100 µL of tick homogenate using a commercially available DNA extraction kit according to the manufacturer’s instructions (Real Genomics DNA extraction kit, Real Biotechnology Corporation, Banqiao City, Taiwan). Elution of extracted DNA was completed using 100 µL of elution buffer provided in the kit. 

Screening for *Rickettsia* spp. was performed by a real-time qPCR assay targeting citrate synthase gene (*gltA*) sequences specific to the SFG and TG of rickettsiae [29]. PCR amplification with Platinum qPCR SuperMix-UDG Mastermix (Invitrogen, Melbourne, Australia) was performed using a cycling protocol comprising one step at 50 °C for 2 min (for Uracil-DNA glycosylase incubation, which prevents contamination with amplicons from previous PCRs), one step at 95 °C for 2 min, and 40 cycles at 95 °C for 10 s and 60 °C for 20 s. *Rickettsia australis* str. Phillips DNA was used as a positive control.

### 4.3. Molecular Identification and Characterization of Detected Rickettsia *spp.*

A selection (*n* = 3) of tick DNA extracts that were *Rickettsia gltA* qPCR positive were examined in more detail to attempt to molecularly identify any *Rickettsia* spp. detected using genes recommended by Fournier et al. [19] for *Rickettsia* identification and classification. To achieve this, the partial fragments of several gene loci were amplified by conventional PCR and sequenced, including (i) a larger fragment of the *Rickettsia gltA* gene [30], (ii) a gene (*ompA*) encoding the rickettsial outer membrane protein A [30], (iii), *ompB* encoding the rickettsial outer membrane protein B [31], (iv) *sca4* encoding a PS120-like-*Rickettsia* protein [32], and (v) a gene (*17kd*) encoding the *Rickettsia* genus-specific 17-kDa outer membrane antigen [30]. Amplicon sequencing was performed by Macrogen (Seoul, Geumcheon-gu, South Korea). 

Analysis of the resulting sequences was performed using SnapGene (GSL Biotech, San Diego, USA) and the NCBI BLAST software (http://blast.ncbi.nlm.nih.gov/Blast.cgi). Amplified sequences from this study are available in Genbank (MT653612-MT653616). To gain more insight into the phylogenetic relationship between the detected *Rickettsia* and those previously described, evolutionary analysis was performed. 

A phylogenetic tree of the trimmed and concatenated sequences (*gltA, ompB, sca4,* and *17kd*) amplified from the three tick DNA extracts was inferred using the concatenated sequences extracted from the available genome sequences for a variety of *Rickettsia* spp. and the maximum likelihood method PhyML 3.0 (www.atgc-montpellier.fr) [33] and Tamura–Nei model [34] as present in Geneious Prime 2020.2.5 (www.geneious.com). A second phylogenetic tree was prepared using the amplified *ompA* sequences from the novel *Rickettsia* and the trimmed deposited sequences for related classified rickettsiae and several uncultured rickettsiae, including those previously amplified from ticks removed from *T. rugosa* [18], using the maxium likelihood method [33] and Tamura–Nei model [34] as present in Geneious Prime. 

## 5. Conclusions

Reptile-associated ticks in Australia are known to be colonized with rickettsiae of the SFG. In this study, the tick *Amblyomma albolimbatum*, taken from the shingleback skink (*Tiliqua rugosa*) in the southern regions of Western Australia in 2016, was shown to contain unique rickettsial DNA most closely related phylogenetically to *Rickettsia gravesii*. 

## Figures and Tables

**Table 1:** *Rickettsia* qPCR screening of *A. albolimbatum ticks* sampled in this study.

Locality/Coordinates	Life Cycle Stage	Ticks (*n*)	*Rickettsia* spp. gltA qPCR *n* (%)
Moulyinning (33.2290° S, 117.9377° E)	Adult (female) Adult (male)	2 2	2 (100) 2 (100)
Wagin (33.3054° S, 117.3474° E)	Adult (male)	3	3 (100)
Yanchep (31.5464° S, 115.6327° E)	Adult (female) Adult (male) Nymph	3 4 1	3 (100) 4 (100) 1 (100)
Esperance (33.8608° S, 121.8896° E)	Adult (female) Adult (male)	1 1	1 (100) 1 (100)
Southern Cross (31.2306° S, 119.3278° E)	Adult (female) Adult (male) Nymph	6 11 1	6 (100) 9 (82) 1 (100)
Yellowdine (31.0656° S, 119.8116° E)	Adult (female) Adult (male) Nymph	2 4 2	2 (100) 3 (75) 2 (100)
Tammin (31.6410° S, 117.4865° E)	Adult (female) Adult (male)	1 5	1 (100) 4 (80)
Youndegin (31.8227° S, 117.2518° E)	Adult (female) Adult (male)	1 2	1 (100) 2 (100)
Doodlakine (31.6020° S, 117.8976° E)	Adult (male)	1	1 (100)
Korbel (31.6082° S, 118.1272° E)	Adult (female) Adult (male)	5 6	5 (100) 5 (100)
	Totals	64	59 (92.2)

## Data Availability

The data presented in this study are openly available in GenBank. For accession number please see above.
